# Identification and Pathogenicity of *Paramyrothecium* Species Associated with Leaf Spot Disease in Northern Thailand

**DOI:** 10.3390/plants11111445

**Published:** 2022-05-29

**Authors:** Patchareeya Withee, Sukanya Haituk, Chanokned Senwanna, Anuruddha Karunarathna, Nisachon Tamakaew, Parichad Pakdeeniti, Nakarin Suwannarach, Jaturong Kumla, Piyawan Suttiprapan, Paul W. J. Taylor, Milan C. Samarakoon, Ratchadawan Cheewangkoon

**Affiliations:** 1 Department of Entomology and Plant Pathology, Faculty of Agriculture, Chiang Mai University, Chiang Mai 50200, Thailand; patchareeya_withee@cmu.ac.th (P.W.); sukanya_hai@cmu.ac.th (S.H.); chanokned.swn@gmail.com (C.S.); anumandrack@yahoo.com (A.K.); nisachon_t@cmu.ac.th (N.T.); parichad_pakdee@cmu.ac.th (P.P.); piyawan.s@cmu.ac.th (P.S.); 2 Research Center of Microbial Diversity and Sustainable Utilization, Chiang Mai University, Chiang Mai 50200, Thailand; suwan.462@gmail.com (N.S.); jaturong_yai@hotmail.com (J.K.); 3 Department of Biology, Faculty of Science, Chiang Mai University, Chiang Mai 50200, Thailand; 4 Innovative Agriculture Research Centre, Faculty of Agriculture, Chiang Mai University, Chiang Mai 50200, Thailand; 5 Faculty of Veterinary and Agricultural Sciences, The University of Melbourne, Parkville, VIC 3010, Australia; paulwjt@unimelb.edu.au

**Keywords:** climate change, diversity, food security, multi-gene phylogeny, new species, plant pathology, taxonomy

## Abstract

Species of *Paramyrothecium* that are reported as plant pathogens and cause leaf spot or leaf blight have been reported on many commercial crops worldwide. In 2019, during a survey of fungi causing leaf spots on plants in Chiang Mai and Mae Hong Son provinces, northern Thailand, 16 isolates from 14 host species across nine plant families were collected. A new species *Paramyrothecium vignicola* sp. nov. was identified based on morphology and concatenated (ITS, *cmdA*, *rpb2*, and *tub2*) phylogeny. Further, *P. breviseta* and *P. foliicola* represented novel geographic records to Thailand, while *P. eichhorniae* represented a novel host record (*Psophocarpus* sp., *Centrosema* sp., *Aristolochia* sp.). These species were confirmed to be the causal agents of the leaf spot disease through pathogenicity assay. Furthermore, cross pathogenicity tests on *Coffea arabica* L., *Commelina benghalensis* L., *Glycine max* (L.) Merr., and *Dieffenbachia seguine* (Jacq.) Schott revealed multiple host ranges for these pathogens. Further research is required into the host–pathogen relationship of *Paramyrothecium* species that cause leaf spot and their management. Biotic and abiotic stresses caused by climate change may affect plant health and disease susceptibility. Hence, proper identification and monitoring of fungal communities in the environment are important to understand emerging diseases and for implementation of disease management strategies.

## 1. Introduction

Plant diseases have a high impact on food security [1] and fungi play a major role in plant diseases [2]. Foliar fungal pathogens severely affect the yield and health of commercial crops [3]. Leaf spots are an early indicator of foliar diseases and may initially occur on the adaxial leaf surfaces and then appear on the abaxial leaf surface.

*Paramyrothecium* species have been frequently identified to cause leaf spot and blight disease on a wide range of vegetables, ornamental plants, and economic crops [4,5,6,7]. Disease symptoms caused by *Paramyrothecium* may also include stem and crown canker and fruit rot [8,9,10]. Lombard et al. [4] designated an epitype for the generic type *Paramyrothecium roridum* (≡*M. roridum*). *Paramyrothecium* species are distinguished from related *Myrothecium* sensu stricto and other myrothecium-like genera by the presence of 1–3 septate, thin-walled setae surrounding the sporodochia. Currently, there are 19 species listed in Index Fungorum (http://www.indexfungorum.org/; accessed on 14 April 2022).

*Paramyrothecium roridum* and *P. foliicola* are well-known pathogens that cause leaf spot or leaf blight and have been reported on many commercial crops and a wide range of hosts, such as soybean, strawberry, and muskmelon [5,8,9]. Rennberger and Keinath [11] isolated *P. foliicola* and *P. humicola* from watermelon and two other cucurbits and confirmed their pathogenicity on watermelons, tomatoes, and southern peas. Aumentado and Balendres [12] reported *P. foliicola* causing crater rot in eggplant and 45 plant species from 21 plant families and were tested for the pathogenicity on detached fruit or leaf assays. Furthermore, *P. foliicola* is pathogenic to cucumber seedlings and watermelon, causing stem canker [13]. Due to the lignicolous nature of the *Paramyrothecium*, they are being used as bio-pesticides for the control of weeds and insects [14,15]. Interestingly, several important secondary metabolites or toxins found in *Paramyrothecium* include trichothecenes macrolides such as roridin, verrucarin, and mytoxin B, which are important for some medicinal and biotechnological applications [16,17,18].

In Thailand, only *P. eichhorniae* has been reported and this was identified as the cause of the leaf blight disease of water hyacinth [19]. The diversity of *Paramyrothecium* species in Thailand is unknown. As a result, surveys and additional research on the distribution of *Paramyrothecium* in Thailand is required. The objective of this study was to identify and describe *Paramyrothecium* spp. from northern Thailand and assess their pathogenicity across a broad range of potential host plant species.

## 2. Results

### 2.1. Symptoms

**Figure 1:**
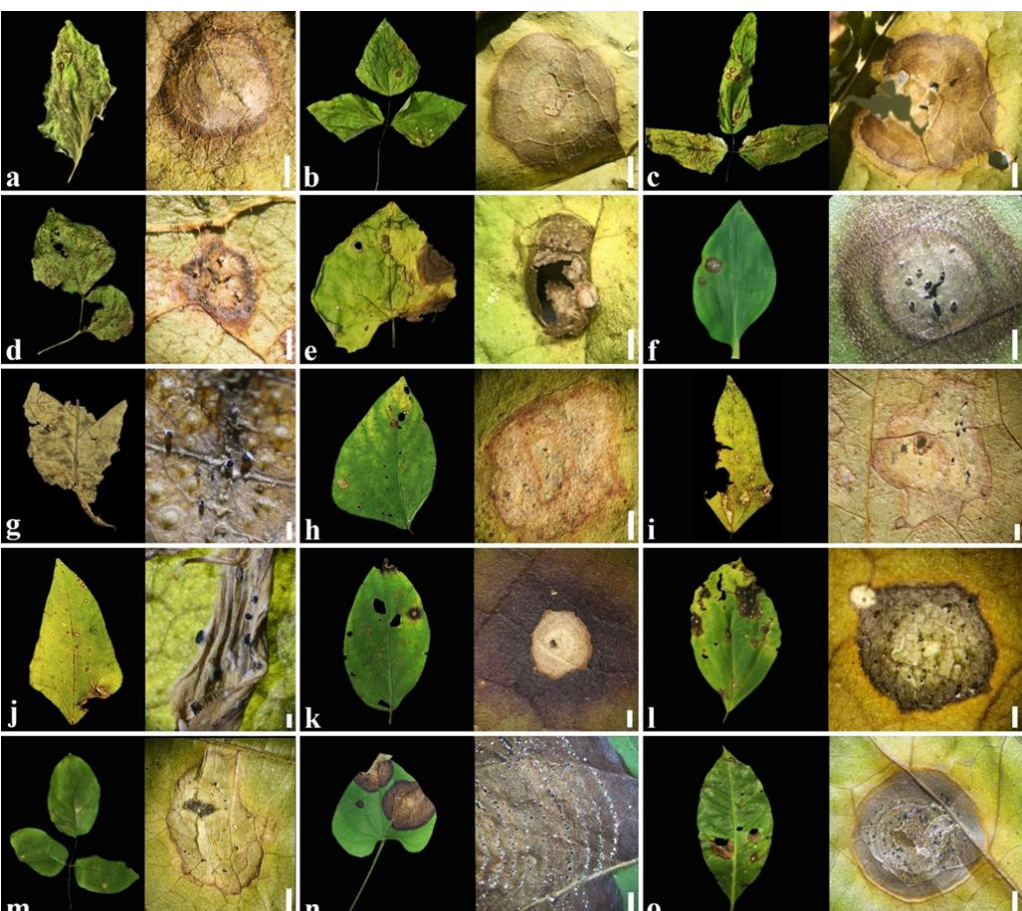
Symptoms on different hosts caused by *Paramyrothecium* (**left**) and sporodochia on the host surface (**right**); (**a**) *Solanum virginianum*; (**b**) *Lablab purpureus*; (**c**) *Psophocarpus* sp.; (**d**,**i**) *Vigna* sp.; (**e**) *Coccinia grandis*; (**f**) *Commelina benghalensis*; (**g**) *Tectona grandis*; (**h**) *Vigna mungo*; (**j**) *Vigna unguiculata*; (**k**) *Oroxylum indicum*; (**l**) *Spilanthes* sp.; (**m**) *Centrosema* sp.; (**n**) *Aristolochia* sp.; (**o**) *Coffea arabica*. Scale bars: (**c**,**g**,j) = 1 mm; (**b**,**d**–**f**,**l**) = 2 mm; (**a**,**i**,**k**) = 4 mm; (**h**,**m**) = 5 mm; (**o**) = 6 mm; (**n**) = 1 cm.

Leaf spots varying in size and shape, depending on the host, were most visible on the upper surface. The leaf spots consisted of small brown spots or necrotic lesions with a dark border, while in older lesions, small sporodochia were visible (Figure 1f,n,o). Necrotic lesions appeared dark gray or black on *Centrosema* sp., *Coccinia grandis*, *Oroxylum indicum*, *Solanum virginianum*, *Tectona grandis*, *Vigna mungo*, *Vigna* sp., and *V. unguiculata* (Figure 1a,d,e,g,h–k,m), and surrounded by a prominent yellow halo on *Lablab purpureus*, *Psophocarpus* sp., and *Spilanthes* sp. (Figure 1b,c,l). Lesions on *Aristolochia* sp., *Coffea arabica*, and *Commelina benghalensis* consisted of light to dark brown concentric rings with a target-like appearance, and small sporodochia that appeared on lower and upper surfaces (Figure 1f,n,o).

### 2.2. Culture Morphology

**Figure 2:**
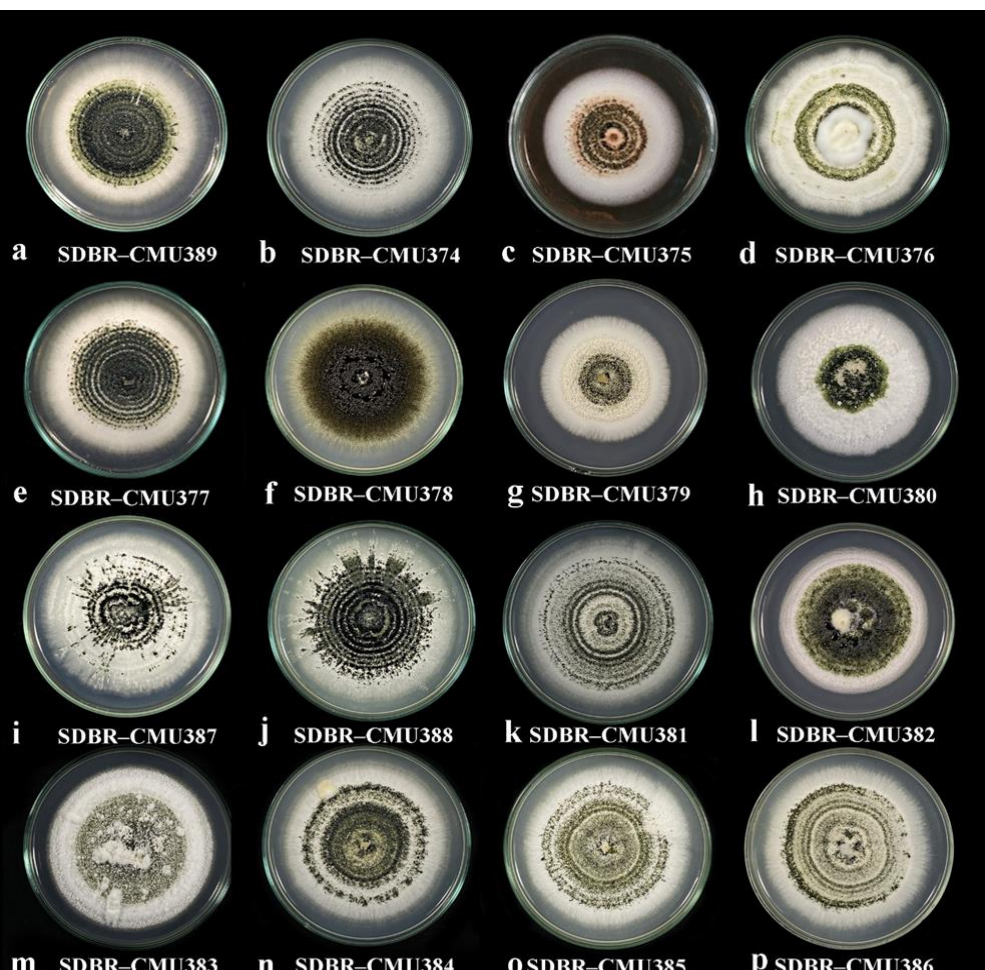
Colonies of *Paramyrothecium* species on PDA after 15 days at 25–30 °C.

Diverse culture characters were observed on PDA at room temperature (25–30 °C) (Figure 2). Eleven isolates of *Paramyrothecium* sp. (SDBR-CMU374, SDBR-CMU375, SDBR-CMU376, SDBR-CMU377, SDBR-CMU378, SDBR-CMU379, SDBR-CMU380, SDBR-CMU382, SDBR-CMU387, SDBR-CMU388, and SDBR-CMU389) (Figure 2a–j,l) formed whitish colonies with entire to slightly undulated margins, radial or in concentric rings with sporodochia, covered with slimy olivaceous green to black conidial masses, while the other four isolates (SDBR-CMU383, SDBR-CMU384, SDBR-CMU385, and SDBR-CMU386) (Figure 2m–p) formed abundant white aerial mycelium with sporodochia forming on the stroma and surface of the medium, covered by slimy olivaceous green to black conidial masses. Isolate SDBR-CMU381 (Figure 2k) produced exudates with brown pigment into the medium.

### 2.3. Phylogenetic Analysis

**Figure 3:**
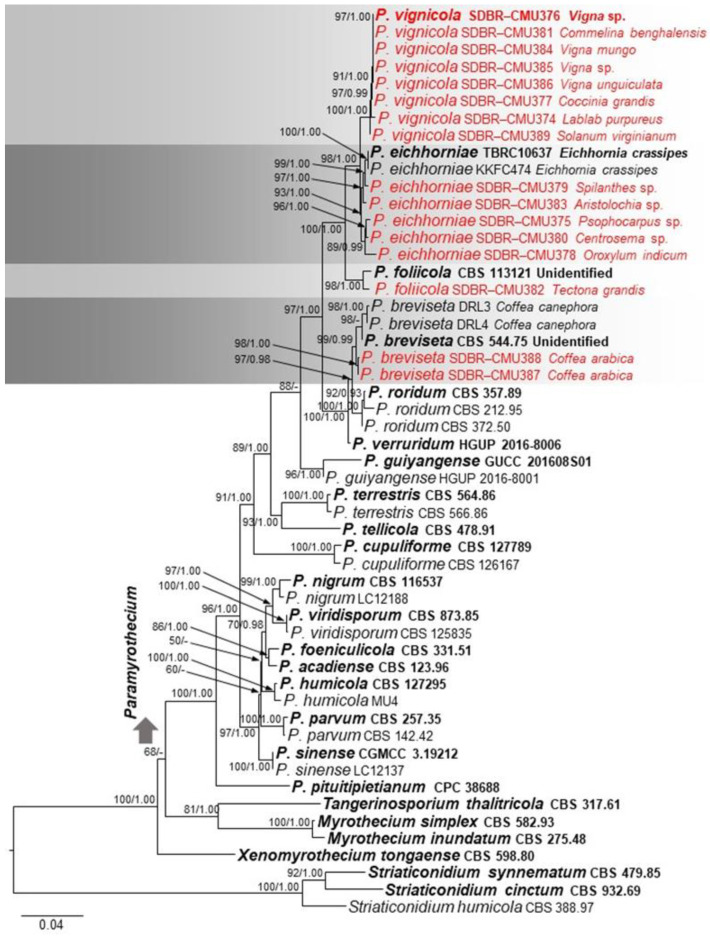
Phylogram generated from maximum likelihood analysis based on combined ITS, *cmdA*, *rpb2*, and *tub2* sequenced data. Fifty-three strains are included in the combined sequence analyses, which comprise 1760 characters with gaps. Single gene analyses were also performed, and topology and clade stability were compared from combined gene analyses. *Striaticonidium cinctum* (CBS 932.69), *S. humicola* (CBS 388.97), and *S. synnematum* (CBS 479.85) are used as the outgroup taxa. The best scoring RAxML tree with a final likelihood value of −8176.4871 is presented. The matrix had 524 distinct alignment patterns. Estimated base frequencies were as follows; A = 0.2266, C = 0.2915, G = 0.2681, T = 0.2138; substitution rates AC = 1.1215, AG = 5.1556, AT = 1.0792, CG = 1.2292, CT = 11.1203, GT = 1.0000; gamma distribution shape parameter α = 0.3855. The bootstrap support (≥50%) of ML and the posterior probability values (≥0.9) of BI analyses are indicated above or below the respective branches. The fungal isolates from this study are indicated in red. The type species are indicated in bold.

The phylogenetic tree topologies of the ML and BI analyses for concatenated ITS, *cmdA*, *rpb2*, and *tub2* were similar. Hence, a phylogenetic tree from ML analyses is used to represent the results of both ML and BI analyses. The dataset comprised 53 taxa with 1760 characters (ITS: 1–542; *cmdA*: 543–824; *rpb2*: 825–1548; *tub2*: 1549–1760), including gaps. The GTR+G+I model was the best-fit model for all loci. The best scoring likelihood tree was selected on the basis of the ML analysis, with a final ML optimization likelihood value of −8176.4871, as shown in Figure 3. Sixteen new isolates were clustered into four distinct clades in *Paramyrothecium* (see the notes).

### 2.4. Taxonomy

Isolates from symptomatic living leaves of different hosts were recognized under *Paramyrothecium* based on taxonomy (Table 1) and multi-gene phylogeny (Figure 3). The morphologies of the *Paramyrothecium* species are described herein.

**Figure 4:**
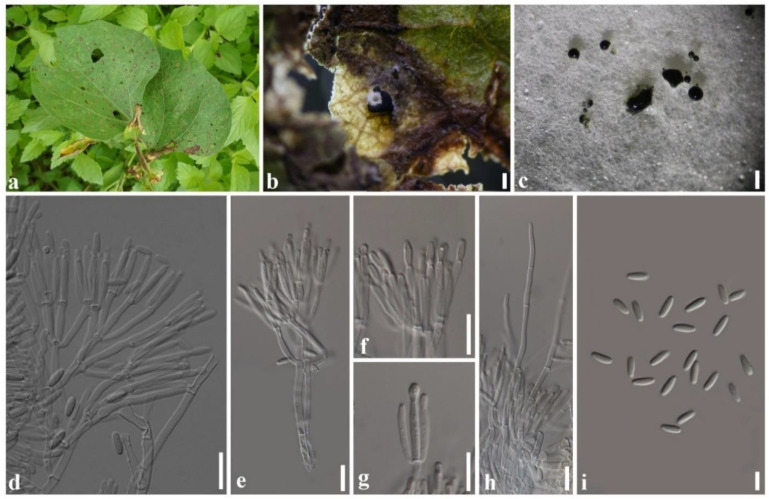
*Paramyrothecium vignicola* (CRC4-H, holotype); (**a**) leaf spot of *Vigna* sp.; (**b**) sporodochia on leaf; (**c**) sporodochial conidiomata on PDA; (**d**,**e**) conidiophores and conidiogenous cells; (**f**,**g**) conidiogenous cells; (**h**) setae; (**i**) conidia. Scale bars: (**b**,**c**) = 1 mm; (**d**–**h**) = 10 µm; (**i**) = 5 µm.

***Paramyrothecium vignicola*** Withee & Cheew., sp. nov. (Figure 4).

Mycobank: MB 843763.

Etymology: Name reflects the host genus Vigna, from which the species was collected.

Holotype: SDBR–CMU376.

Description: Sexual morph: unknown. Asexual morph: *Conidiomata* sporodochial, stromatic, superficial, cupulate, scattered or gregarious, oval or irregular in outline, (60–)90–300(–385) µm diam, (70–)140–180(–200) µm deep, with a white to creamy setose fringe surrounding an olivaceous green agglutinated slimy mass of conidia. *Stroma* poorly developed, hyaline. *Setae* arising from the stroma thin-walled, hyaline, 3–8-septate, straight becoming sinuous above the apical septum, 80–155 μm long, 2–3 μm wide, tapering to an acutely rounded apex. *Conidiophores* arising from the basal stroma, consisting of a stipe and a penicillately branched conidiogenous apparatus; stipes unbranched, hyaline sometimes covered by a green mucoid layer, septate, smooth, 40–60 × 2–3 µm; primary branches aseptate, unbranched, smooth, 10–26 × 2–3 µm ($\bar{x}$ = 18 × 3 µm, *n* = 20); secondary branches aseptate, unbranched, smooth, 10–17 × 2–3 µm ($\bar{x}$ = 13 × 3 µm, *n* = 20); terminating in a whorl of 3–6 conidiogenous cells; conidiogenous cells phialidic, cylindrical to subcylindrical, hyaline, smooth, straight to slightly curved, 11–16 × 1–3 µm ($\bar{x}$ = 13 × 2 µm, *n* = 20), with conspicuous collarettes and periclinal thickenings. *Conidia* aseptate, hyaline, smooth, cylindrical to ellipsoidal, 5–7 × 1–3 µm ($\bar{x}$ = 6 × 2 µm, *n* = 20), rounded at both ends.

Culture characteristics: Colonies on PDA, dense, circular, flattened, slightly raised, floccose, white aerial mycelium, radiating with concentric ring of sporodochia forming, covered by slimy olivaceous green to black conidial masses.

Material examined: Thailand, Mae Hong Son Province, on living leaf of *Vigna* sp. (*Fabaceae*), 11 September 2019, N. Tamakaew, CRC4-H (holotype), ex-type living culture SDBR-CMU376; *ibid.*, on living leaf of *Solanum virginianum* (*Solanaceae*), 11 September 2019, N. Tamakaew, CRC1-H, living culture SDBR-CMU389; *ibid.*, on living leaf of *Lablab purpureus* (*Fabaceae*), 11 September 2019, N. Tamakaew, CRC2-H, living culture SDBR-CMU374; *ibid.*, on living leaf of *Coccinia grandis* (*Cucurbitaceae*), CRC6-H, living culture SDBR-CMU377; Chiang Mai province, on living leaf of *Commelina benghalensis* (*Commelinaceae*), 20 November 2019, P. Withee, CRC14-H, living culture SDBR-CMU381; *ibid.*, on living leaf of *Vigna mungo* (*Fabaceae*), 5 December 2019, N. Tamakaew, CRC144-H, living culture SDBR-CMU384; *ibid.*, on living leaf of *Vigna* sp. (*Fabaceae*), CRC145-H, living culture SDBR-CMU385; *ibid.*, on living leaf of *Vigna unguiculata* (*Fabaceae*), 10 February 2020, P. Withee, CRC146-H, living culture SDBR–CMU386.

Notes: Based on ITS, *cmdA*, *rpb2* and *tub2* phylogeny (Figure 3) and *cmdA* and *tub2* (data not shown), *Paramyrothecium foliicola* formed two distinct clades. The clade with *Paramyrothecium foliicola* type (CBS 113121) was treated as the *Paramyrothecium* sensu stricto. Eight of the new strains clustered with eight previously described *Paramyrothecium* strains (as *P*. *foliicola*) and formed a well-supported clade (100% BS/1.00 PP) (*Paramyrothecium* sensu lato) closely related to *P*. *eichhorniae* and *P*. *foliicola* (Figure 3). Based on morphology and phylogeny, we introduce a new species to accommodate taxa in *P. foliicola* sensu lato. *Paramyrothecium vignicola* differs from *P. eichhorniae* and *P*. *foliicola* with longer setae (up to 155 μm vs. up to 120 μm and up to 100 μm). The conidia of *P. vignicola* (5–7 × 1–3 µm) are slightly larger than those of *P*. *eichhorniae* (5–6.5 × 1.5–2.5 µm) [20] and *P*. *foliicola* (5–6 × 1–2 µm) [4]. *Paramyrothecium vignicola* differs from other *Paramyrothecium* species by its 3–8-septate, thin-walled setae surrounding the sporodochia. In BLAST searches of NCBI GenBank, the closest matches of the sequences are *Paramyrothecium*: *P*. *foliicola* (CBS 11321) with 98.98% similarity in ITS sequence, 93.89% similarity in *cmdA*. *P*. *vignicola*, 96.32% in *tub2* with *P*. *foliicola* (CBS 11321). Based on phylogenetic evidence and morphological differences, *P*. *vignicola* is a new species.

**Figure 5:**
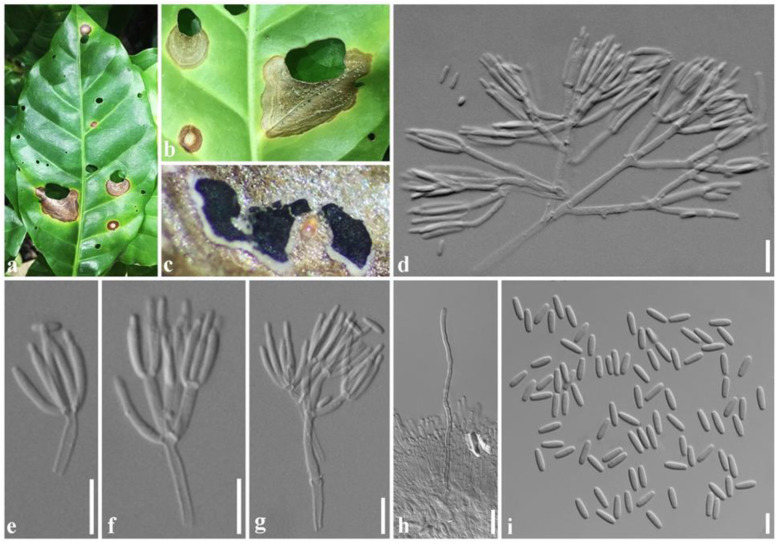
*Paramyrothecium breviseta* (CRC13-H); (**a**,**b**) leaf spot of *Coffea arabica*; (**c**) sporodochia on leaf; (**d**) conidiophores and conidiogenous cells; (**e**–**g**) conidiogenous cells; (**h**) setae; (**i**) conidia. Scale bars: (**d**) = 20 µm; (**e**–**h**) = 10 µm; (**i**) = 5 µm.

***Paramyrothecium breviseta*** L. Lombard & Crous, in Lombard et al., Persoonia 36: 207 (2016) (Figure 5).

Description: Sexual morph: unknown. Asexual morph: *Conidiomata* sporodochial, stromatic, cupulate, superficial, scattered or rarely gregarious, oval or irregular in outline, 135–790 µm diam, 9–15 µm deep, with a white setose fringe surrounding an olivaceous green to black agglutinated slimy mass of conidia. *Setae* arising from the stroma thin-walled, hyaline, 1–5-septate, straight to flexuous, 25–120 μm long, 2–3 μm wide, tapering to an acutely rounded apex. *Conidiophores* arising from the basal stroma, consisting of a stipe and a penicillately branched conidiogenous apparatus; stipes unbranched, hyaline, septate, smooth, 6–9 × 2–4 µm; primary branches aseptate, unbranched, smooth, 12–24 × 3–4 µm ($\bar{x}$ = 18 × 3 µm, *n* = 20); secondary branches aseptate, unbranched, smooth, 10–17 × 2–4 µm ($\bar{x}$ = 12 × 3 µm, *n* = 20); terminating in a whorl of 3–6 conidiogenous cells; conidiogenous cells phialidic, cylindrical to subcylindrical, hyaline, smooth, straight to slightly curved, 6–11 × 1–2 µm ($\bar{x}$ = 9 × 2 µm, *n* = 20), with conspicuous collarettes and periclinal thickenings. *Conidia* aseptate, hyaline, smooth, cylindrical to ellipsoidal, 5–7 × 1–2 µm ($\bar{x}$ = 6 × 2 µm, *n* = 20), rounded at both ends.

Culture characteristics: Colonies on PDA, dense, circular, flattened, slightly raised, floccose, white aerial mycelium, radiating with concentric ring of sporodochia forming, covered by slimy olivaceous green to black conidial masses.

Material examined: Thailand, Chiang Mai, on living leaf of *Coffea arabica* (*Rubiaceae*), 20 November 2019, R. Cheewangkoon and P. Withee, CRC13-H, living culture SDBR-CMU387; *ibid.*, CRC12-H, living culture SDBR-CMU388.

Notes: Phylogenetically, SDBR-CMU387 and SDBR-CMU388 formed a well-supported clade closely related to *Paramyrothecium breviseta* L. Lombard & Crous (Figure 2). *Paramyrothecium breviseta* was collected on an unknown substrate in India [4] and in this study, we collected *P. breviseta* from *Coffea arabica* (*Rubiaceae*) in Chiang Mai Province. The morphology of the fresh specimen is similar to that described by Lombard et al. [4], but the conidia (5–7 × 1–2 vs. 4–5 × 1–2 µm) and setae (25–120 × 2–3 vs. 25–40 × 2–3 µm) are longer. However, this is the first host report of leaf spot causing *P*. *breviseta* on *C. arabica* in Thailand.

**Figure 6:**
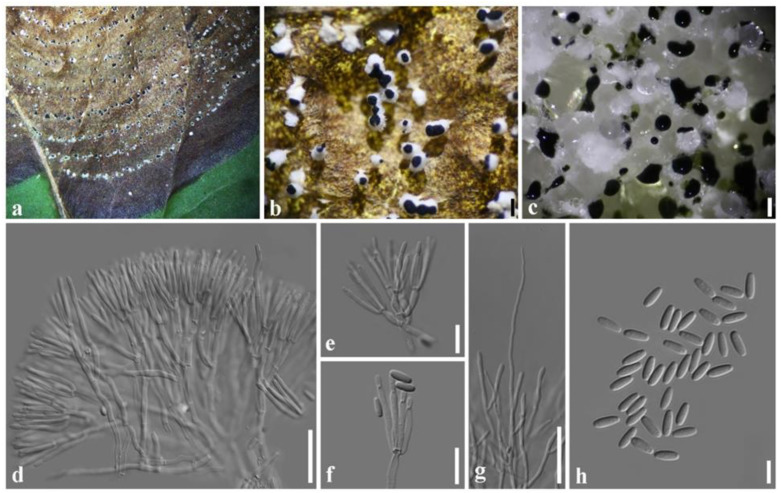
*Paramyrothecium eichhorniae* (CRC143); (**a**) leaf spot of *Aristolochia* sp.; (**b**) sporodochia on leaf; (**c**) sporodochial conidiomata on PDA; (**d**) sporodochia; (**e**,**f**) conidiogenous cells; (**g**) setae; (**h**) conidia. Scale bars: (**b**,**c**) = 1 mm; (**d**,**g**) = 20 µm; (**e**,**f**) = 10 µm; (**h**) = 5 µm.

***Paramyrothecium eichhorniae*** J. Unartngam, A. Unartngam & U. Pinruan, in Pinruan et al., Mycobiology 50: 17 (2022) (Figure 6).

Description: Sexual morph: unknown. Asexual morph: *Conidiomata* sporodochial, stromatic, superficial, cupulate, scattered or gregarious, oval or irregular in outline, (60–)70–250(–500) µm diam, (60–)70–270(−370) µm deep, with a white setose fringe surrounding an olivaceous green to dark green slimy mass of conidia. *Setae* arising from the stroma thin-walled, hyaline, 1–5-septate, straight to flexuous, 60–120 μm long, 2–3 μm wide, tapering to an acutely rounded apex. *Conidiophores* arising from the basal stroma, consisting of a stipe and a penicillately branched conidiogenous apparatus; stipes unbranched, hyaline, septate, smooth, 15–40 × 2–3 µm; primary branches aseptate, unbranched, smooth, 10–17 × 2–3 µm ($\bar{x}$ = 12 × 3 µm, *n* = 20); secondary branches aseptate, unbranched, smooth, 7–14 × 2–3 µm ($\bar{x}$ = 10 × 3 µm, *n* = 20); terminating in a whorl of 3–6 conidiogenous cells; conidiogenous cells phialidic, cylindrical to subcylindrical, hyaline, smooth, straight to slightly curved, 11–17 × 2–3 µm ($\bar{x}$ = 14 × 2 µm, *n* = 20), with conspicuous collarettes and periclinal thickenings. *Conidia* aseptate, hyaline, smooth, cylindrical to ellipsoidal, 5–7 × 1–2 µm ($\bar{x}$ = 6 × 2 µm, *n* = 20), rounded at both ends.

Culture characteristics: Colonies on PDA, entire to slightly undulated margins, with sporodochia forming on the surface of the medium, covered by slimy olivaceous green to black conidial masses.

Material examined: Thailand, Mae Hong Son Province, on living leaf of *Psophocarpus* sp. (*Fabaceae*), 11 September 2019, N. Tamakaew, CRC3-H, living culture SDBR-CMU375; *ibid.*, on living leaf of *Oroxylum indicum* (*Bignoniaceae*), 11 September 2019, N. Tamakaew, CRC8-H, living culture SDBR-CMU378; *ibid.*, on living leaf of *Spilanthes* sp. (*Asteraceae*), 11 September 2019, N. Tamakaew, CRC148-H, living culture SDBR-CMU379; *ibid.*, on living leaf of *Centrosema* sp. (*Fabaceae*), 11 September 2019, N. Tamakaew, CRC11-H, living culture SDBR-CMU380; Chiang Mai province, on living leaf of *Aristolochia* sp. (*Aristolochiaceae*), January 2020, P. Suttiprapan, CRC143-H, living culture SDBR-CMU383.

Note: Based on multigene phylogeny, five isolates in this study clustered with *Paramyrothecium eichhorniae*, which was associated with water hyacinth (*Eichhornia crassipes*) and recently described from Thailand [10]. Morphologically, the conidiogenous cells of our collections are similar to those of the holotype of *P. eichhorniae*. However, the conidia of *P. eichhorniae* in this study are thinner than reported by Pinruan et al. [19] (5–7 × 1–2 µm vs. 5–6.5 × 1.5–2.5 µm) and have more septa in setae than the holotype (1–5 vs. 1–3 septate). This is the first report of *P. eichhorniae* on *Psophocarpus* sp., *Centrosema* sp., and *Aristolochia* sp. from Thailand.

**Figure 7:**
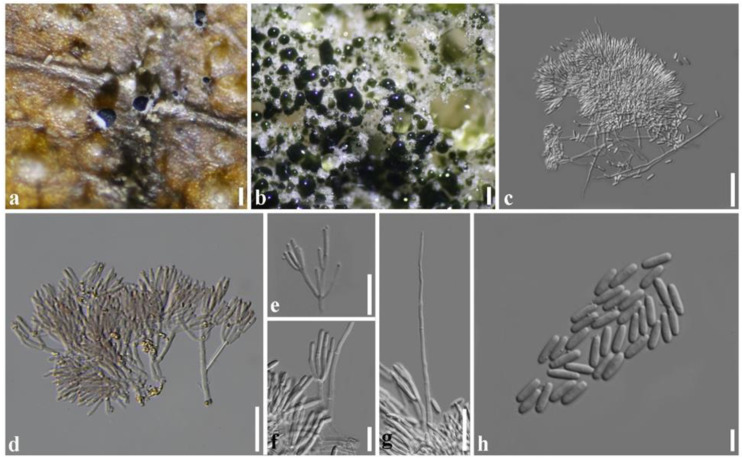
*Paramyrothecium foliicola* (CRC15); (**a**) sporodochia on leaves of *Tectona grandis* (**b**) sporodochial conidiomata on PDA; (**c**,**d**) sporodochia (**e**,**f**) conidiogenous cells; (**g**) setae; (**h**) condia. Scale bars: (**a**) = 500 µm; (**b**) = 1 mm; (**c**) = 30 µm; (**d**,**g**) = 20 µm; (**h**) = 5 µm.

***Paramyrothecium foliicola*** L. Lombard & Crous, in Lombard et al., Persoonia 36: 209 (2016) (Figure 7).

Description: Sexual morph: unknown. Asexual morph: *Conidiomata* sporodochial, stromatic, superficial, cupulate, scattered or gregarious, oval or irregular in outline, (60–)100–170(–245) µm diam, (70–)140–165(–200) µm deep, with a white to creamy setose fringe surrounding an olivaceous green agglutinated slimy mass of conidia. *Stroma* poorly developed, hyaline. *Setae* arising from the stroma thin-walled, hyaline, 1–4(–8)-septate, straight becoming sinuous above the apical septum, 35–175 μm long, 2–3 μm wide, tapering to an acutely rounded apex. *Conidiophores* arising from the basal stroma, consisting of a stipe and a penicillately branched conidiogenous apparatus; stipes unbranched, hyaline sometimes covered by a green mucoid layer, septate, smooth, 20–75 × 2–4 µm; primary branches aseptate, unbranched, smooth, (10–)17–21(–26) × 2–3(–4) µm ($\bar{x}\;$= 15 × 3 µm, *n* = 20); secondary branches aseptate, unbranched, smooth, (7–)9–17(–19) × 2–3(–4) µm ($\bar{x}\;$= 14 × 3 µm, *n* = 20); terminating in a whorl of 3–6 conidiogenous cells; conidiogenous cells phialidic, cylindrical to subcylindrical, hyaline, smooth, straight to slightly curved, 10–17 × 1–3 µm ($\bar{x}\;$= 13 × 2 µm, *n* = 20), with conspicuous collarettes and periclinal thickenings. Conidia aseptate, hyaline, smooth, cylindrical to ellipsoidal, 5–8 × 1–3 µm ($\bar{x}\;$= 7 × 2 µm, *n* = 20), rounded at both ends.

Culture characteristics: Colonies on PDA, abundant white aerial mycelium with sporodochia forming on the aerial mycelium and surface of the medium, covered by slimy olivaceous green to black conidial masses.

Materials examined: Thailand, Chiang Mai, on living leaf of *Tectona grandis* (*Lamiaceae*), 20 November 2019, P. Withee, CRC15-H, living culture SDBR-CMU382.

Notes: Based on our phylogenetic analysis (Figure 3), SDBR-CMU382 isolates were clustered with *Paramyrothecium foliicola*. The morphology of our collection (CRC15-H) is similar to that of *P. foliicola* described by Lombard et al. [4]. However, our collection has longer conidiophores (20–75 × 2–4 vs. 15–25 × 2–3 µm) and more septa in setae (1–4(–8) vs. 1–3 septate), conidiogenous cells (10–17 × 1–3 vs. 8–14 × 1–2 µm) and conidia (5–8 × 1–3 vs. 5–6 × 1–2 µm). This may be due to distribution, environment, and morphological variability within the species. This is the first report of *P. foliicola* from *Tectona grandis* in Thailand.

### 2.5. Pathogenicity Test and Cross Pathogenicity

**Figure 8:**
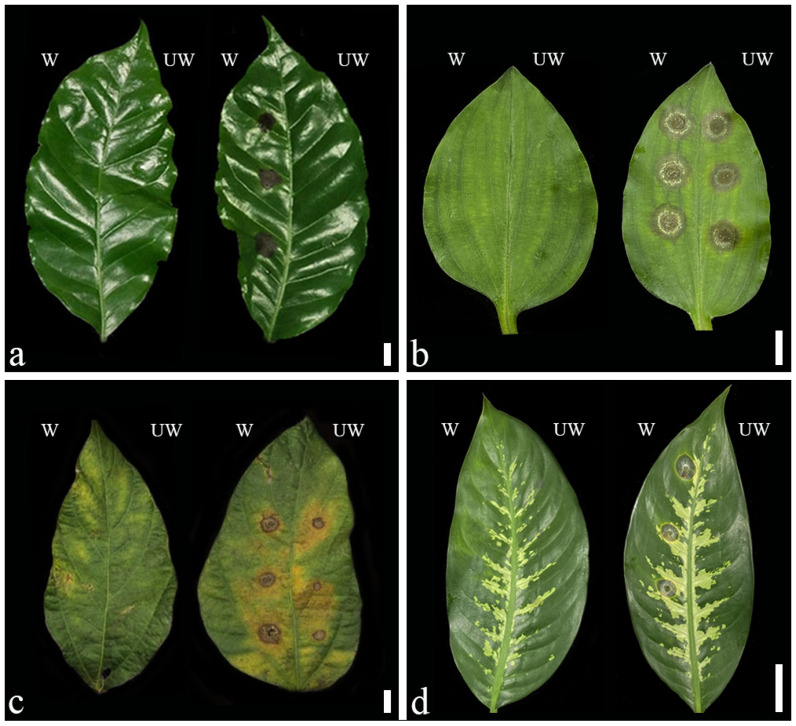
Pathogenicity test (**a**,**b**) and cross pathogenicity (**c**,**d**); Control (left); (**a**) *Paramyrothecium brevista* on *Coffea arabica*; (**b**) *P*. *vignicola* on *Commelina benghalensis*; (**c**) *P. vignicola* on *Glycine max*; (**d**) *P. vignicola* on *Dieffenbachia seguine*; (w) wound and (uw) unwound. Scale bars: (**a**–**c**) = 1 cm; (**d**) = 6 cm.

Koch’s postulates confirmed that all the fungal isolates were able to cause disease in unwounded leaves of *Commelina benghalensis* and *Glycine max* (Figure 8b,c). The SDBR–CMU383 isolate infected all inoculated plants and was highly aggressive on most, except for *C. benghalensis*. No infection was observed in the unwounded inoculation of *Coffea arabica* and *Dieffenbachia seguine* (Figure 8a,d). Leaves receiving sterilized distilled water remained healthy. The fungi were re-isolated from the diseased leaf tissues in each experiment, and each isolated fungus was identical to the inoculated fungus. Further, Koch’s postulates confirmed that all isolates of *Paramyrothecium vignicola*, *P. breviseta*, *P. eichhorniae*, and *P. foliicola* were pathogenic to their original host plants. Cross pathogenicity tests showed that all isolates infected inoculated (wounded) *C. arabica*, *C. benghalensis*, *G. max*, and *D. seguine* leaves (Table 2). The symptoms showed light to dark brown and irregular to round lesions, which had scattered olive-colured sporodochia and dark exudates of spore masses (Figure 8).

## 3. Discussion

The new species *Paramyrothecium vignicola* was described using morphology and multi-gene phylogeny and the host range included *Solanum virginianum* (*Solanaceae*), *Lablab purpureus* (*Fabaceae*), *Coccinia grandis* (*Cucurbitaceae*), *Commelina benghalensis* (*Commelinaceae*), *Vigna* sp., *V. mungo*, and *V. unguiculata* (*Fabaceae*). Multi-gene phylogeny using ITS, *cmdA*, *rpb2*, and *tub2* sequence data clearly identified *P. eichhorniae*, *P. vignicola*, *P. breviseta*, and *P. foliicola* as distinct species within *Paramyrothecium*. Further, multi-gene phylogeny precisely demonstrated the species delineation of *Paramyrothecium*.

The pathogenicity assays showed that *P. vignicola*, *P. breviseta*, *P. eichhorniae*, and *P. foliicola* isolated from different hosts from different locations in northern Thailand can all cause leaf spot disease on different host families, including *Rubiaceae*, *Fabaceae*, *Commelinaceae*, and *Araceae*. However, the disease severity was related to the plant species and inoculation methods, where *Paramyrothecium* spp. could not cause disease in *Coffea arabica* and *Dieffenbachia seguine* without wounding. Wounding involves the breakage of the plant’s first barrier of defense; cuticle and epidermal cells. The tissue then becomes more susceptible to the pathogens. Some species cannot infect non-wounded leaves, hence they are weakly aggressive on these hosts [15]. On the other hand, *Commelina benghalensis* and *Glycine max* were susceptible to all isolates. These results are similar to those of Rennberger and Keinath [11] and Aumentado and Balendres [12], in which *Parammyrothecium* species were able to infect original and non-original hosts within the same family (host shift ability) and different families (host jump ability).

For species diversity and distribution, more gene studies and more reference sequences are needed to resolve the species boundaries of *Paramyrothecium*. Field inspections are needed to confirm the importance of this pathogen and prove that diseases associated with *Paramyrothecium* species are threats to economic crops in Thailand. The information on the spread of related species to new areas is necessary as climate change may enable saprotrophic fungi to switch their nutritional mode across a wider host range, even if an area is predicted to be at risk from an introduced pathogen. It may be the case that few of the susceptible host species are present in this predicted area [26], so for the risk to be realized, climate change should also favor the migration of susceptible species or increase the susceptibility of the resident hosts.

*Paramyrothecium* leaf spot occurs in commercially important plants (*Coffea arabica*, *Tectona grandis*, *Vigna mungo*, and *V. unguiculata*) as well as on non-commercial plants (*Aristolochia* sp., *Centrosema* sp., *Coccinia grandis*, *Commelina benghalensis*, *Lablab purpureus*, *Oroxylum indicum*, *Psophocarpus* sp., *Solanum virginianum*, *Spilanthes* sp., and *Vigna* sp.). In cross pathogenicity assays, all the isolates from host plants could induce the disease on non-original hosts. *Paramyrothecium* species can stay in non-commercial plants, and they can infect commercially important crops. Hence, *Paramyrothecium* leaf spot disease has the potential to be an emerging fungal disease in Thailand. Thus, more research on *Paramyrothecium* is required for epidemiology studies and management strategies in agriculture, horticulture, and plantation forestry.

## 4. Materials and Methods

### 4.1. Sample Collection

Symptomatic plant leaves were collected from fields or forests in different locations in northern Thailand. The name of the host, location, and collection dates were recorded. Specimens were taken to the lab, and infected leaves were examined directly using the stereo microscope (Zeiss Stemi 305) to observe the fungal structures (sporodochia). Symptomatic leaves without fungal structures were also incubated in moist chambers (Petri dishes containing moist filter paper). Leaves were inspected daily for Paramyrothecium-like fungi.

### 4.2. Fungal Isolation and Taxonomic Description

Fungal structures on leaf samples were mounted in lactic acid and photographed under a light microscope (Axiovision Zeiss Scope-A1). Measurements were made with the Tarosoft (R) Image Frame Work program (Tarosoft, Bangkok, Thailand). The fungi were isolated using the single spore isolation technique [27]. Cultures were plated onto fresh PDA and incubated at 25–30 °C in daylight to promote sporulation. Cultural characteristics were observed after 14 days. The specimens were deposited in the fungal collection library at the Department of Entomology and Plant Pathology (CRC), Faculty of Agriculture, Chiang Mai University, Chiang Mai, Thailand. Pure fungal isolates were deposited in the Culture Collection of the Sustainable Development of Biological Resources Laboratory (SDBR), Faculty of Science, Chiang Mai University, Chiang Mai, Thailand.

### 4.3. DNA Extraction, Amplification, and Analyses

Fungal mycelia were grown on PDA at 25–30°C for 7 days and DNA was extracted by using the DNA Extraction Mini Kit (FAVORGEN, Ping-Tung, Taiwan) following the manufacturer’s instructions. DNA amplifications were performed by polymerase chain reaction (PCR). The relevant primer pairs used in this study are listed in Table 3.

The quality of PCR amplification was confirmed on 1% agarose gel electrophoresis and viewed under ultraviolet light, and the sizes of amplicons were determined against a HyperLadderTM I molecular marker (BIOLINE). Further purification of PCR products was performed using the PCR Clean-up Gel Extraction NucleoSpin **^®^** Gel and PCR Clean-Up Kit (Macherey-Nagel, Düren, Germany). The purified PCR fragments were sent to the 1st Base Company (Kembangan, Selangor, Malaysia). The obtained nucleotide sequences were deposited in GenBank.

Sequences were assembled using SeqMan 5.00 and the closely related taxa for newly generated sequences were selected from GenBank^®^ based on BLAST searches of the NCBI nucleotide database (http://blast.ncbi.nlm.nih.gov/; accessed on 4 March 2022). The reference nucleotide sequences of representative genera in *Stachybotriaceae* are in Table 4. The individual gene sequences were initially aligned by MAFFT version 7 [33] (http://mafft.cbrc.jp/align-ment/server/; accessed on 4 March 2022) and improved manually where necessary in BioEdit v.7.0.9.1 [34]. The final alignment of the combined multigene dataset was analyzed and inferred the phylogenetic trees based on maximum likelihood (ML) and Bayesian inference (BI) analyses. The ML analyses were carried out on RAxML-HPC2 on XSEDE (v. 8.2.8) [35,36] via the CIPRES Science Gateway platform [37]. Maximum likelihood bootstrap values (BS) equal or greater than 50% are defined above each node. The BI analyses were performed by MrBayes on XSEDE, MrBayes 3.2.6 [38] via the CIPRES Science Gateway. Bayesian posterior probabilities (PP) [39,40] were determined by Markov Chain Monte Carlo Sampling (BMCMC). Six simultaneous Markov chains were run from random trees for 2,000,000 generations, and trees were sampled every 100th generation. The run was stopped when the standard deviation of split frequencies was reached at less than 0.01. The first 20% of generated trees representing the burn-in phase of the analysis were discarded, and the remaining trees were used for calculating PP in the majority rule consensus tree. The Bayesian posterior probabilities (BYPP) equal to or greater than 0.9 are defined above the nodes. The phylogenetic tree was visualized in FigTree v.1.4.3 [41] and edited in Adobe Illustrator CC 2021 version 23.0.3.585 and Adobe Photoshop CS6 version 13.0. (Adobe Systems, New York, USA).

### 4.4. Pathogenicity Tests and Cross Pathogenicity

Koch’s postulates were used to confirm the pathogenicity of all the isolates on their original hosts. Cross pathogenicity of all the isolates was performed in healthy leaves of selected economically important plants in northern Thailand, including *Coffea arabica* (*Rubiaceae*) and *Glycine max* (*Fabaceae*) and widespread herbaceous plants including *Commelina benghalensis* (*Commelinaceae*) and *Dieffenbachia seguine* (*Araceae*). Healthy leaves were surface disinfected with 70% ethanol, washed two times with sterile distilled water, and air-dried under laminar flow. Conidial suspensions (10^6^ conidia/mL) were prepared for all fungal isolates in sterile distilled water. The conidia (10 μL of spore suspension) were placed on the upper surface of the leaves. In addition, the leaves were also wounded before inoculation. The upper epidermis was wounded approximately 2 cm from the mid-vein by pricking with a sterile needle to about 1 mm depth. Three wounds were made for each leaf, vertically on each side of the mid-vein. Control leaves received drops of sterile distilled water. All inoculated leaves were placed in a moist chamber at 25–30 °C under daylight condition. After 7 days, symptoms were recorded, compared, and confirmed with the original morphology and molecular relationships.

## 5. Conclusions

Leaf spots caused by *Paramyrothecium* spp. were isolated from commercially important plants (*Coffea arabica*, *Tectona grandis*, *Vigna mungo*, and *V. unguiculata*), and non-commercial plants (*Aristolochia* sp., *Centrosema* sp., *Coccinia grandis*, *Commelina benghalensis*, *Lablab purpureus*, *Oroxylum indicum*, *Psophocarpus* sp., *Solanum virginianum*, *Spilanthes* sp., and *Vigna* sp.) in northern Thailand. Based on morphology and concatenated (ITS, *cmdA*, *rpb2*, and *tub2*) phylogeny, *P. vignicola*, *P. breviseta, P. eichhorniae*, and *P. foliicola* were identified. The pathogenicity of each isolate was proven using Koch’s postulates. The pathogenicity assay revealed that all the isolates can cause the leaf spot disease. Interestingly, cross pathogenicity assay proved the ability of all 16 isolates to cause the disease on a wide range of hosts.

## Figures and Tables

**Table 1:** Synopsis of *Paramyrothecium* type species.

Species	Host	Location	Conidiophores (µm)	Conidiogenous Cells (µm)	Conidia (µm)	Setae (µm)	References
*Paramyrothecium acadiense*	*Tussilago farfara*	Canada	9–14 × 2–2.5	–	0–1-septate, 5.5–16.5 × 1.5–2.5	–	[20]
*P. breviseta*	unknown	India	6–9 × 2–4	6–11 × 1–2	aseptate, 4–5 × 1–2	1–3-septate, 25–40 × 2–3	[4]
*P. cupuliforme*	Soil	Namibia	15–25 × 2–4	4–11 × 1–3	aseptate, 6–8 × 1–2	1–3-septate, 45–90 × 2–3	[4]
*P. eichhorniae*	*Eichhornia crassipes*	Thailand	15–40 × 2–3	(8–)11–17(–20) × 2–3	aseptate,5– 6.5 × 1.5–2.5	1–3-septate, 40–120 × 2–3	[19]
*P. foeniculicola*	*Foeniculum vulgare*	Netherlands	7–17 × 2–3	6–16 × 1–2	aseptate, 5–7 × 1–2	–	[4]
*P. foliicola*	Decaying leaf	Brazil	15–25 × 2–3	8–14 × 1–2	aseptate, 5–6 × 1–2	1–3-septate, 60–100 × 2–3	[4]
*P. guiyangense*	Soil	China	10−60 × 1−3	8−18 × 1.6−2.7	aseptate, 6.6−9.0 × 2−3	1−3-septate, 60−120 × 1−3	[21]
*P. humicola*	Soil	USA	12–22 × 2–3	8–13 × 1–3	aseptate, 6–7 × 1–2	1–2-septate, 55–65 × 2–3	[4]
*P. lathyri*	*Lathyrus tuberosus*	Russia	5–10 × 2–3.5	5–10 × 2–3	aseptate, (8–)9 (–10) × 2(–2.5)	3–10-septate, up to 300 × 3–4	[22]
*P. nigrum*	Soil	Spain	25–45 × 2–4	8–13 × 1–2	aseptate, 5–6 × 1–2	1–3-septate, 60–100 × 2–3	[4]
*P. parvum*	*Viola* sp.	UK	12–26 × 2–4	7–23 × 1–2	aseptate, 4–5 × 1–2	–	[4]
*P. pituitipietianum*	*Grielum humifusum*	South Africa	20–35 × 3–4	20–35 × 3–4	aseptate, (7–)9–10(–12) × (2–)2.5	7–10-septate, 100–300 × 4–5	[23]
*P. roridum*	*Gardenia* sp.	Italy	15–40 × 2–4	7–33 × 2–3	aseptate, (5–)6.5–7.5(–8) × 2	1–3(–4)-septate, 60–100 × 2–6	[4]
*P. salvadorae*	*Salvadora persica*	Namibia	20–40 × 3–4	8–15 × 2–2.5	aseptate, (8–)10–12 (–13) × 2–2.5	5–10- septate, 100–200 × 2.5–3	[24]
*P. sinense*	Rhizosphere soils of *Poa* sp.	China	20–30 × 2–3	7–16 × 1–3	aseptate, 6–7 × 2–3	1–3-septate, 45–90 × 1–3	[25]
*P. tellicola*	Soil	Turkey	15–30 × 2–4	7–17 × 1–3	aseptate, (7–)7.5–8.5 (–9) × 1–3	1–3-septate, 45–80 × 2–3 μm	[4]
*P. terrestris*	Soil	Turkey	15–30 × 2–3	7–12 × 2–3	aseptate, (7–)7.5–8.5 (–10) × 1–3	1–3-septate, 35–70 × 2–3	[4]
*P. verruridum*	Soil	China	20−40 × 1.5−2.5	12−20 × 1.7−2.7	aseptate, 6.8−7.8 × 2−2.7	1−3-septate, 40−120 × 2−3	[21]
*P. vignicola*	*Vigna* sp.	Thailand	40–60 × 2–3	11–16 × 1–3	aseptate, 5–7 × 1–3 µm	3–8-septate, 80–155 × 2–3	This study
*P. viridisporum*	Soil	Turkey	15–35 × 2–3	6–12 × 3–5	aseptate, 3–5 × 2 µm	1–3-septate, 60–140 × 2–3	[4]

**Table 2:** Pathogenicity test and cross pathogenicity of *Paramyrothecium* species on original hosts and other plant species.

Species		Isolates	Plant Hosts							
	Original Host		*Coffea arabica*		*Commelina benghalensis*		*Glycine max*		*Dieffenbachia seguine*	
			w	uw	w	uw	w	uw	w	uw
*P. vignicola*	*Vigna* sp.	SDBR-CMU376 ^T^	+	-	+	+	+	+	+	-
	*Lablab purpureus*	SDBR-CMU374	+	-	+	+	+	+	+	-
	*Coccinia grandis*	SDBR-CMU377	+	-	+	+	+	+	+	-
	*Commelina benghalensis*	SDBR-CMU381	+	-	+	+	+	+	+	-
	*Vigna mungo*	SDBR-CMU384	+	-	+	+	+	+	+	-
	*Vigna* sp.	SDBR-CMU385	+	-	+	+	+	+	+	-
	*Vigna unguiculata*	SDBR-CMU386	+	-	+	+	+	+	+	-
	*Solanum virginianum*	SDBR-CMU389	+	-	+	+	+	+	+	-
*P. brevista*	*Coffea arabica*	SDBR-CMU387	+	-	+	+	+	+	+	-
	*Coffea arabica*	SDBR-CMU388	+	-	+	+	+	+	+	-
*P. eichhorniae*	*Psophocarpus* sp.	SDBR-CMU375	+	-	+	+	+	+	+	-
	*Oroxylum indicum*	SDBR-CMU378	+	-	+	+	+	+	+	-
	*Spilanthes* sp.	SDBR-CMU379	+	-	+	+	+	+	+	-
	*Centrosema* sp.	SDBR-CMU380	+	-	+	+	+	+	+	-
	*Aristolochia* sp.	SDBR-CMU383	+	-	-	-	+	+	+	-
*P. foliicola*	*Tectona grandis*	SDBR-CMU382	+	-	+	+	+	+	+	-

Note: (-) No symptoms (+) Symptoms (w) wound and (uw) unwound.; Superscript “^T^” indicates type species.

**Table 3:** Gene regions and primer sequences used in this study.

Gene Regions	Primers	Sequence (5′→3′)	Length (bp)	References
ITS	ITS5 ITS4	GGA AGT AAA AGT CGT AAC AAG G TCC GCT TAT TGA TAT GC	ca. 600	[28]
cmdA	CAL–228F CAL–737R CAL2Rd	GAG TTC AAG GAG GCC TTC TCC C CAT CTT TCT GGC CAT GG TGR TCN GCC TCD CGG ATC ATC TC	CAL–228F–CAL–737R: 470–570 CAL–228F–CAL2Rd: 680–745	[29,30]
rpb2	RPB2–5F RPB2–7cR	GAY GAY MGW GAT CAY TTY GG CCC ATR GCT TGY TTR CCC AT	ca. 1000	[31]
*tub2*	Bt2a Bt2b	GGT AAC CAA ATC GGT GCT TTC ACC CTC AGT GTA GTG ACC CTT GGC	ca. 320	[32]

**Table 4:** Taxa used in the phylogenetic analyses and their corresponding GenBank numbers.

Species	Isolate No.	Substrate	Location	GenBank Accession Numbers			
				ITS	*cmdA*	*rpb2*	*tub2*
*Myrothecium inundatum*	CBS 275.48 ^T^	On decaying pileus of *Russula nigricans*	England	KU846452	KU846435	–	KU846533
*M. simplex*	CBS 582.93 ^T^	On decaying agaric	Japan	KU846456	KU846439	–	KU846537
*Paramyrothecium acadiense*	CBS 123.96 ^T^ = DAOMC 221473 = UAMH 7653	On leaves of *Tussilago farfara*	Canada	KU846288	–	KU846350	KU846405
*P*. *breviseta*	CBS 544.75 ^T^	unknown	India	KU846289	KU846262	KU846351	KU846406
	DRL3	On leaves of *Coffea canephora*	China	MT853067	MT897897	–	MT897899
	DRL4	On leaves of *C. canephora*	China	MT853068	MT897898	–	MT897900
	**SDBR-CMU387**	**On living leaf of** ***C. arabica***	**Thailand**	**MZ373251**	**OM810407**	**ON033773**	**OM982450**
	**SDBR-CMU388**	**On living leaf of** ***C. arabica***	**Thailand**	**MZ373252**	**OM810408**	**ON033774**	**OM982451**
*P. cupuliforme*	CBS 127789 ^T^	On surface soil in desert	Namibia	KU846291	KU846264	KU846353	KU846408
	CBS 126167	On surface soil in desert	Namibia	KU846290	KU846263	KU846352	KU846407
*P. eichhorniae*	TBRC 10637 ^T^	On leaf of *Eichhornia crassipes*	Thailand	MT973996	MT975319	MT975317	MT977540
	KKFC 474	On leaf of *E.crassipes*	Thailand	MT973995	MT975318	MT977541	MT975316
	**SDBR-CMU375**	**On living leaf of *Psophocarpus* sp.**	**Thailand**	**MZ373241**	**OM810411**	**ON033781**	**ON033770**
	**SDBR-CMU378**	**On living leaf of unidentified plant**	**Thailand**	**MZ373246**	**OM810414**	**ON033782**	**ON033772**
	**SDBR-CMU379**	**On living leaf of unidentified plant**	**Thailand**	**MZ373247**	**OM810415**	**ON033783**	**ON033768**
	**SDBR-CMU380**	**On living leaf of *Centrosema* sp.**	**Thailand**	**MZ373250**	**OM810416**	**ON033784**	**ON033771**
	**SDBR-CMU383**	**On living leaf of *Aristolochia* sp.**	**Thailand**	**MZ373255**	**OM810418**	**ON033785**	**ON033769**
*P. foeniculicola*	CBS 331.51 ^T^ = IMI 140051	On leaf sheath *Foeniculum vulgare*	The Netherlands	KU846292	–	KU846354	KU846409
*P. foliicola*	CBS 113121 ^T^ = INIFAT C02/104 ^T^	On rotten leaf of unknown host	Brazil	KU846294	KU846266	–	KU846411
	**SDBR-CMU382**	**On decaying leaf of *Tectona grandis***	**Thailand**	**MZ373254**	**–**	**ON033775**	**OM982452**
*P. guiyangense*	GUCC 201608S01 ^T^	From soil	China	KY126418	KY196193	–	KY196201
	HGUP 2016–8001	From soil	China	KY126417	KY196192	–	KY196200
*P. humicola*	CBS 127295 ^T^	from tallgrass prairie soil	USA	KU846295	–	KU846356	KU846412
	MU4	On leaf of *Citrullus lanatus*	USA	MN227389	MN593629	MN397959	MN398054
*P. nigrum*	CBS 116537 ^T^ = AR 3783	From soil	Spain	KU846296	KU846267	KU846357	KU846413
	LC12188	Rhizosphere soils of *Poa* sp.	China	MK478871	MK500252	MK500261	MK500269
*P. parvum*	CBS 257.35 ^T^ = IMI 140049	On *Viola* sp.	UK	KU846298	–	KU846359	KU846415
	CBS 142.42 = IMI 155923 = MUCL 7582	From dune sand	France	KU846297	KU846268	KU846358	KU846414
*P. pituitipietianum*	CPC38688 ^T^	On stems of *Grielum humifusum*	South Africa	MW175358	MW173100	–	MW173139
*P. roridum*	CBS 357.89 ^T^	On *Gardenia* sp.	Italy	KU846300	KU846270	KU846361	KU846417
	CBS 212.95	From water	The Netherlands	KU846299	KU846269	KU846360	KU846416
	CBS 372.50 = IMI 140050	On twig of *Coffea* sp.	Colombia	KU846301	KU846271	KU846362	KU846418
*P. sinense*	CGMCC3.19212 ^T^ = LC12136	Rhizosphere soils of *Poa* sp.	China	MH793296	MH885437	MH818824	MH793313
	LC12137	Rhizosphere soils of *Poa* sp.	China	MH793295	MH885436	MH818822	MH793312
*P. tellicola*	CBS 478.91 ^T^	From soil	Turkey	KU846302	KU846272	KU846363	KU846419
*P. terrestris*	CBS 564.86 ^T^	From soil under *Lycopersicon esculentum*	Turkey	KU846303	KU846273	KU846364	KU846420
	CBS 566.86	From soil beneath *Helianthus annuus*	Turkey	KU846305	KU846275	KU846366	KU846422
*P. verruridum*	HGUP 2016–8006 ^T^	From soil	China	KY126422	KY196197	–	KY196205
*P. vignicola*	**SDBR-CMU389**	**On living leaf of *Solanum virginianum***	**Thailand**	**MZ373239**	**OM810409**	**ON033776**	**ON009013**
	**SDBR-CMU374**	**On living leaf of *Lablab purpureus***	**Thailand**	**MZ373240**	**OM810410**	**ON033777**	**ON009014**
	**SDBR-CMU376** **^T^**	**On living leaf of *Vigna* sp.**	**Thailand**	**MZ373242**	**OM810412**	**ON033778**	**ON009015**
	**SDBR-CMU377**	**On living leaf of *Coccinia grandis***	**Thailand**	**MZ373244**	**OM810413**	**ON033779**	**ON009016**
	**SDBR-CMU381**	**On living leaf of *Commelina benghalensis***	**Thailand**	**MZ373253**	**OM810417**	**ON033780**	**ON009017**
	**SDBR-CMU384**	**On living leaf of *Vigna mungo***	**Thailand**	**MZ373256**	**OM810419**	**ON033786**	**–**
	**SDBR-CMU385**	**On living leaf of *Vigna* sp.**	**Thailand**	**MZ373257**	**OM810420**	**ON033787**	**ON009018**
	**SDBR-CMU386**	**On living leaf of** ***V. unguiculata***	**Thailand**	**MZ373258**	**OM810421**	**ON033788**	**ON009019**
*P. viridisporum*	CBS 873.85 ^T^	From soil	Turkey	KU846308	KU846278	KU846369	KU846425
	CBS 125835	Rhizosphere soils of bunchgrass	USA	KU846310	KU846280	KU846371	KU846427
*Striaticonidium cinctum*	CBS 932.69 ^T^	From agricultural soil	The Netherlands	KU847239	KU847216	KU847290	KU847329
*S. humicola*	CBS 388.97	From soil in tropical forest	Papua New Guinea	KU847241	KU847217	KU847291	KU847331
*S. synnematum*	CBS 479.85 ^T^	From leaf of unknown palm	Japan	KU847242	KU847218	KU847292	KU847332
*Tangerinosporium thalictricola*	CBS 317.61 ^T^ = IMI 034815	On *Thalictrum flavum*	UK	KU847243	KU847219	–	KU847333
*Xenomyrothecium tongaense*	CBS 598.80 ^T^	On dead thallus of *Halimeda* sp.	Tonga	KU847246	KU847221	KU847295	KU847336

Note: CBS: Culture collection of the Centraalbureau voor Schimmelcultures, Fungal Biodiversity Centre, Utrecht, The Netherlands; CGMCC: China General Microbiological Culture Collection Center; CPC: Collection of P.W. Crous; DAOMC: The Canadian Collection of Fungal Cultures; GUCC: Guizhou University Culture Collection, Guiyang, China; HGUP: Herbarium of Guizhou University, Plant Pathology, China; IMI: International Mycological Institute, CABI-Bioscience, Egham, Bakeham Lane; INIFAT: INIFAT Fungus Collection, Ministerio de Agricultura Habana; KKFC: Kasetsart.Kamphaengsaen Fungal Collection, Thailand; LC: Collection of Lei Cai, Institute of Microbiology, Chinese Academy of Sciences, Beijing, China; MUCL: Mycothèque de l’Université Catholique de Louvian, Belgium; SDBR-CMU: the Culture Collection of the Sustainable Development of Biological Resources Laboratory, Faculty of Science, Chiang Mai University, Chiang Mai, Thailand; TBRC: Thailand Bioresource Research Center, Thailand. Species obtained in this study are in bold. Superscript “^T^” indicates type species and “–” represents the absence of sequence data in GenBank.

## Data Availability

Publicly available datasets were analyzed in this study. These data can be found here: https://www.ncbi.nlm.nih.gov/ (access on 30 June 2022).
